# Stent graft placement in anterior tibial artery pseudoaneurysm in a patient with type I neurofibromatosis: A case report

**DOI:** 10.1097/MD.0000000000032447

**Published:** 2022-12-30

**Authors:** Jonghun Woo, Jae Myeong Lee, Jongjoon Shim

**Affiliations:** a Department of Radiology, Soonchunhyang University Bucheon Hospital, Bucheon, Gyeeonggi-do, Korea

**Keywords:** anterior tibial artery, neurofibromatosis, stent graft

## Abstract

Vascular involvement manifests as aneurysms, stenosis, and arteriovenous malformations in patients with type I neurofibromatosis (NF-I). Aneurysms are rare; however, the renal artery is the most common site. Herein, we report a rare case of stent graft placement in an anterior tibial artery (ATA) pseudoaneurysm in a patient with NF-I.

**Patient concerns:**

A 52-year-old woman with NF-1 was admitted to the emergency room with painful swelling in the left lower leg. At presentation, the patient’s blood pressure was 100/60 mmHg and the hemoglobin level was 9 g/dL.

**Diagnoses:**

Computed tomography scan revealed a small aneurysm arising from the left ATA and an adjacent large hematoma.

**Intervention:**

Stent graft placement was performed to treat ATA pseudoaneurysm.

**Outcomes:**

After stent graft placement, the aneurysm disappeared and the distal flow was patent through the ATA.

**Lessons:**

Stent graft placement should be considered as another option for endovascular treatment in patients in whom coil embolization or surgery cannot be performed.

## 1. Introduction

Neurofibromatosis type I (NF-I) is an autosomal dominant disorder characterized by café au lait macules, benign neurofibroma, and iris hamartomas.^[1]^ The prevalence of vascular involvement in NF-I is known to range from 0.4% to 6.4%.^[1–3]^ Vascular involvement mainly appears in the form of aneurysms, stenosis, and arteriovenous malformation, and the renal artery is known to be the most commonly involved site.^[2,3]^ However, extremity involvement is very rare.^[1]^ A total of 3 cases of NF-related tibial artery aneurysms have been reported and all were treated surgically.^[4–6]^ In general, for tibial artery pseudoaneurysms or ruptures caused by trauma, coil embolization is the most common endovascular treatment.^[7,8]^ Stent grafts are used for wide-necked pseudoaneurysms; however, a stent graft that can be used for peripheral intervention has a diameter of 5 mm or more, which is too large for use in the tibial artery. In such cases, a coronary device should be used.^[7,8]^

To the best of our knowledge, there have been no reports of stent graft placement in patients with tibial artery pseudoaneurysms caused by NF-I. Herein, we report the first case of anterior tibial artery (ATA) pseudoaneurysm management using a stent graft in a patient without a posterior tibial artery (PTA) or a peroneal artery.

## 2. Case Report

A 54-year-old woman presented to the emergency room with a painful swelling in the left lower leg that had persisted for 3 days. She had been diagnosed with neurofibromatosis 7 years previously. At the time of admission, the patient’s left leg was severely swollen, her blood pressure was 100/60 mm Hg, and hemoglobin level was 9.0 g/dL.

**Figure 1.**
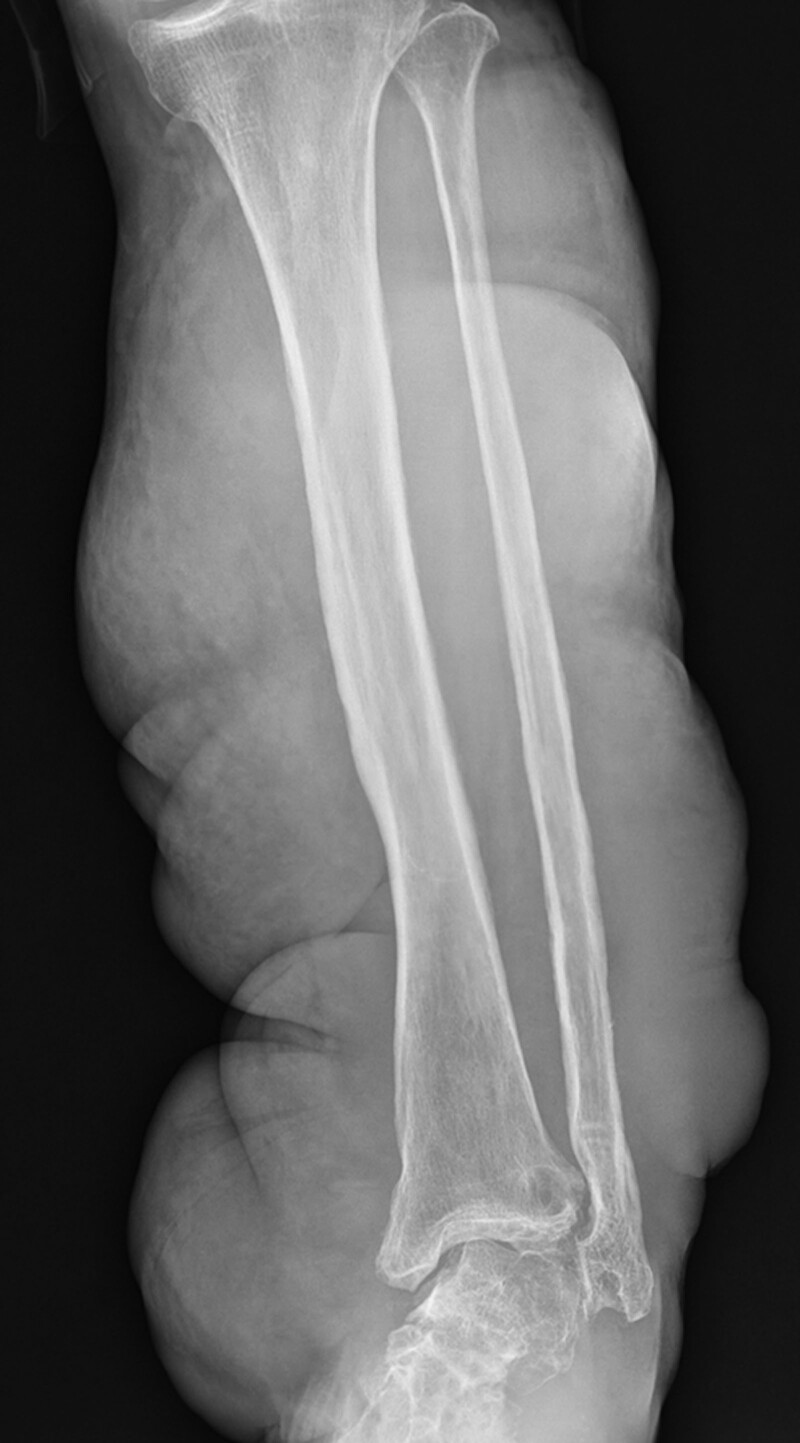
Left leg radiograph showing massive enlargement of soft tissues with cortical irregularity of the tibia and fibula.

**Figure 2.**
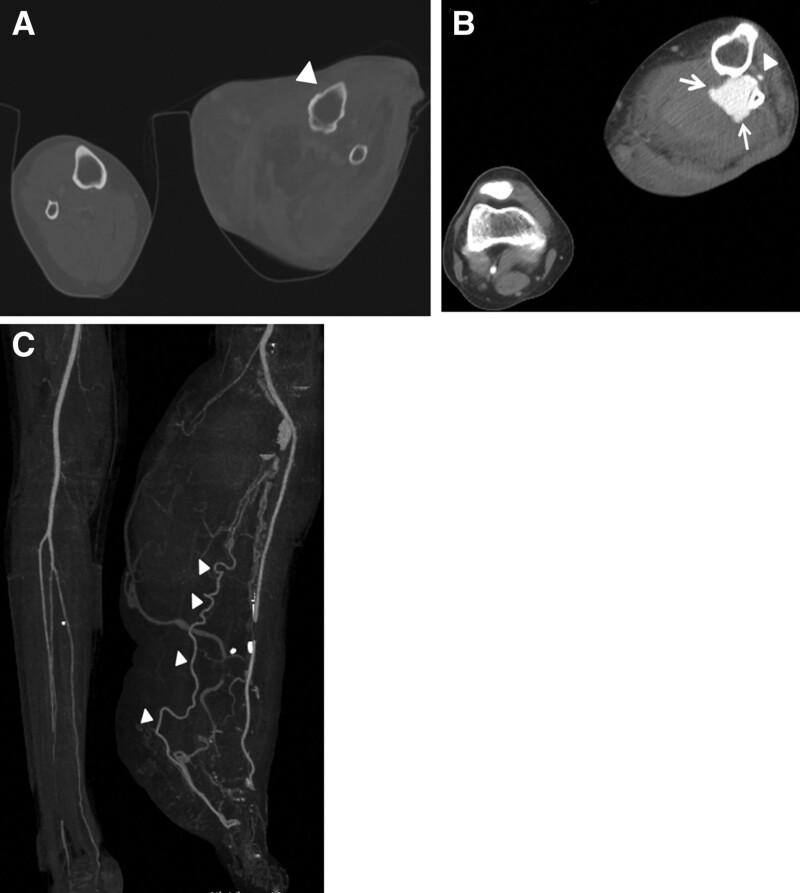
(A) CT bone setting images showing cortical thinning and irregularity (arrowhead) of the left tibia and fibula compared to the right side. (B) Axial CT image showing a pseudoaneurysm (arrow) in the left proximal ATA (arrowhead) and adjacent hematoma. (C) 3D reconstruction CT image in which the PTA and peroneal artery are not visible, and the foot and leg flow is maintained by the collateral flow of the ATA (arrowhead). ATA = anterior tibial artery, CT = computed tomography.

Radiographic imaging performed in the emergency room showed severe swelling of the soft tissue of the left leg and irregularity of the bony cortex (Fig. 1). Computed tomography (CT) revealed a 4.0 × 3.8 cm aneurysm in the proximal portion of the ATA. The diameter of the ATA in this area was approximately 5 mm, and the PTA or peroneal artery was not clearly visible (Fig. 2). The findings of contrast extravasation were not clear, but there was a hematoma around the area and the aneurysm wall was irregular, so the possibility of rupture was high; therefore, we decided to implement interventional treatment.

**Figure 3.**
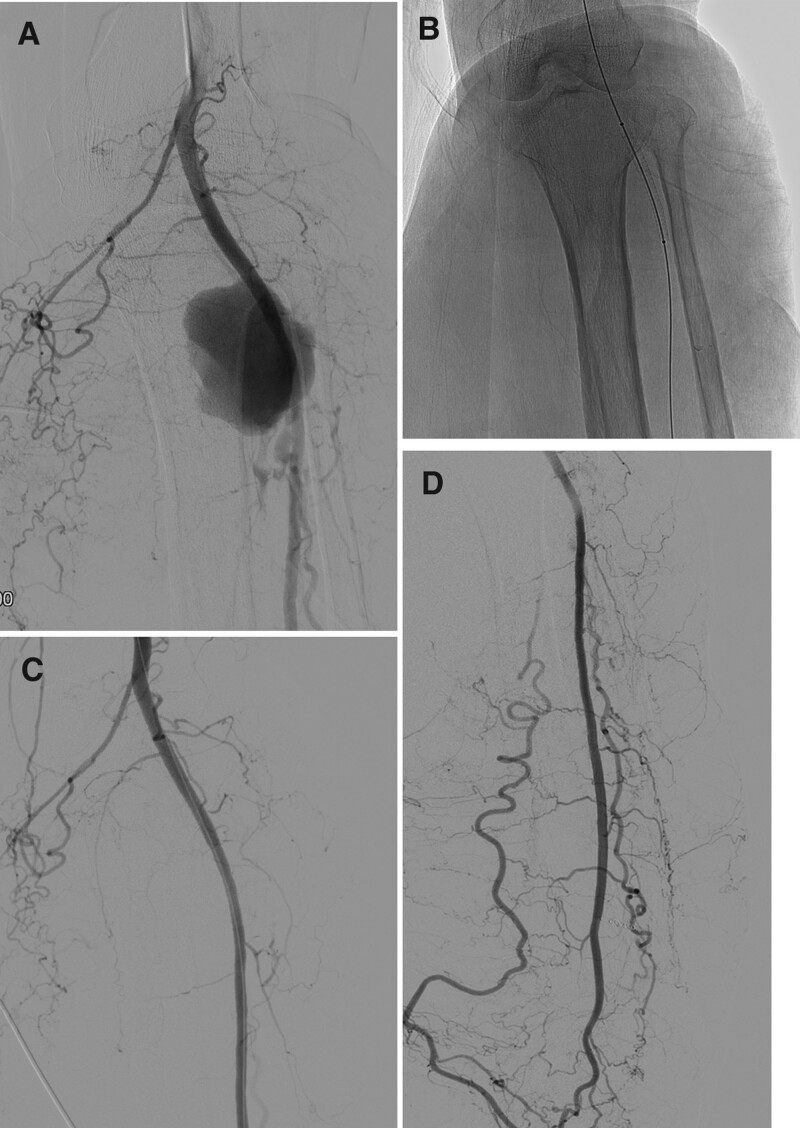
(A) Left common femoral artery angiogram showing pseudoaneurysm with irregular margin in the left proximal ATA, and unclear contrast extravasation. (B, C) A 5-mm stent graft was placed on the left proximal ATA, and the aneurysm disappeared, showing a patent ATA flow. (D) Distal ATA flow is patented, and the flow of the foot and lower leg is maintained by the collateral. ATA = anterior tibial artery.

The right common femoral artery was punctured under US guidance and a 5Fr. short sheath (Terumo, Tokyo, Japan) was inserted. Catheterization of the left superficial femoral artery was attempted by using a 5Fr. Cobra catheter (Terumo, Tokyo, Japan) and 0.035-inch hydrophilic guidewire (Terumo, Tokyo, Japan). On the left SFA angiogram, a 4-cm aneurysm was observed in the proximal portion of the left ATA. However, the tibioperoneal trunk, PTA, and peroneal artery could not be identified. Instead, the ATA diameter in this area was thickened to approximately 5 mm, and collaterals reconstructed from the ATA were seen in the left leg and foot. As the ATA was the only blood vessel supplying the left leg and foot, coil embolization was not possible. The ATA was thick; therefore, we decided to perform stent-graft placement. After changing to a 6Fr. long sheath (Flexor Raabe guiding sheath, COOK, Bloomington, IN), the sheath tip was placed in the left common femoral artery. The wire was then passed through the aneurysm site along the catheter and entered into the distal ATA. A 5 mm × 5 cm Viabahn stent-graft (W.L. Gore & Associates, Flagstaff, AZ) along the wire was installed in the proximal ATA with the aneurysm. Subsequently, the angiogram showed that the aneurysm had disappeared, the distal ATA flow was patent, and the blood supply to the foot through the collateral was well-maintained (Fig. 3).

**Figure 4.**
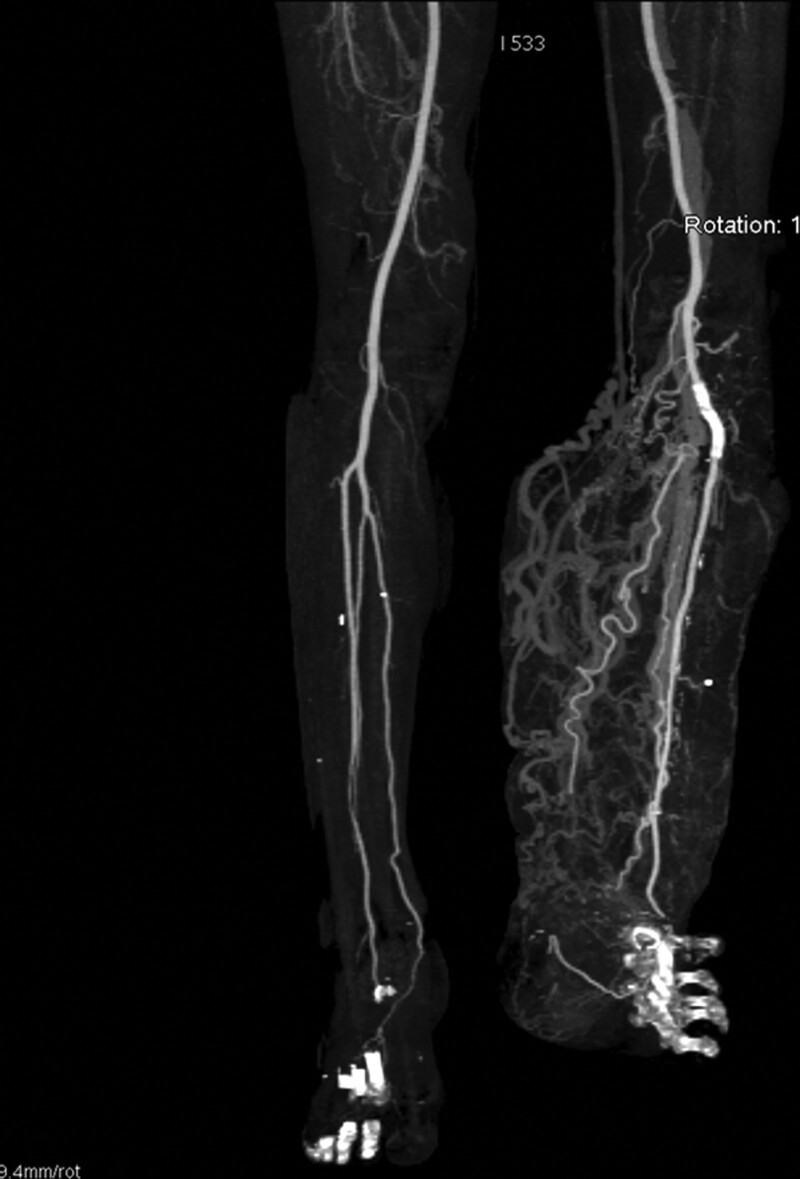
FU CT performed 6 months later showed patent ATA flow through a stent, and the aneurysm had disappeared. ATA = anterior tibial artery, CT = computed tomography.

After the intervention, blood transfusion was performed to restore the reduced hemoglobin level, vital signs were stable, leg swelling gradually decreased, and the patient was discharged a week later. CT performed 6 months later showed that the stent was patent, and the hematoma around the stent had disappeared (Fig. 4).

## 3. Discussion

NF-I is an autosomal dominant disorder characterized primarily by hamartomatous and neoplastic proliferation of peripheral and central nervous system cells due to mutations in the NF-1 gene located on chromosome 17.^[1–3]^ In cases with vascular involvement, the prevalence is known to be 0.4–6.4%, and it mainly appears in the form of aneurysms, stenosis, and arteriovenous malformations.^[1–3]^ Three types of vascular changes (intimal, aneurysmal, and fusocellular) have been reported by Reubi.^[9]^ Salyer and Salyer^[10]^ hypothesized that the proliferation, degenerative changes, and healing process of Schwann cells within the arterial wall are the causes of various vascular abnormalities. However, the mechanisms underlying this phenomenon remain unclear. According to Oderich et al,^[1]^ among vascular abnormalities, the renal artery is the most common site of involvement, and stenosis is more common than aneurysm (41%). They also reported that aneurysms of the carotid, vertebral, or cerebral artery occurred in 19% of cases, and abdominal aortic coarctation or aneurysms occurred in 12% of cases. Peripheral artery lesions are rare, and Lin et al^[11]^ reported that 2% of NF-1 patients had cardiovascular abnormalities, of which only 0.7% had peripheral artery abnormalities.

Tibial artery aneurysms related to NF-I are very rare, so there are few reported cases, and most cases were treated surgically.^[4–6]^ However, in our case, the deformity of the leg with the aneurysm was severe, and the possibility of bleeding during surgery was high due to multiple collaterals, so endovascular treatment was performed. However, since exact endovascular treatment has not been established, the procedure was performed according to the treatment of peripheral artery aneurysms associated with trauma.

The below-knee arteries were the ATA, PTA, and peroneal arteries. If a pseudoaneurysm or rupture is caused by a traumatic injury to one vessel, coil embolization is usually performed. This is because, even if one blood vessel is blocked, arterial flow to the leg or foot can be maintained because there are collaterals from the other 2 blood vessels. Therefore, embolization is difficult when only one blood vessel is present, as in our case. Iliac and femoral artery stenting have become common procedures; however, below-knee artery stenting remains controversial. This is because stent occlusion due to thrombosis in the early stage and neointimal hyperplasia in the late stage after stent placement can occur because the arteries below the knee have a small diameter and slow flow. However, in our case, the thickness of the blood vessel was relatively large (5 mm), and the flow was fast; therefore, there were no complications, such as thrombus, after stent placement. The patient’s condition was well maintained without stent occlusion, even at 6 months follow-up.

In conclusion, tibial artery aneurysms in patients with NF-I are rare, and most are treated surgically. In cases with NF-I-related tibial artery aneurysms, no endovascular treatment has been reported, but in cases with trauma-related tibial aneurysms, coil embolization is usually performed rather than stent grafting due to vessel size. However, as in our case, if the vessel size is sufficient, stent graft placement can be an effective treatment for tibial artery aneurysms caused by NF-I and may be an alternative option when surgery or endovascular coil embolization is not possible.

## Author contributions

**Conceptualization:** Jae Myeong Lee, Jongjoon Shim.

**Funding acquisition:** Jae Myeong Lee.

**Resources:** Jae Myeong Lee.

**Supervision:** Jae Myeong Lee.

**Writing—original draft:** Jonghun Woo

**Writing—review and editing:** Jonghun Woo, Jongjoon Shim.
